# Supplementary material for: Novel Nanosized Spinel MnCoFeO4 for Low-Temperature Hydrocarbon Oxidation
Source: Nanomaterials (Basel). 2022 Nov 4;12(21):3900. doi: 10.3390/nano12213900 (PMC9653678; doi:10.3390/nano12213900)
Supplement: Supplementary file 1 [file nanomaterials-12-03900-s001.zip › nanomaterials-2009740-supplementary.pdf]

Supplementary Materials

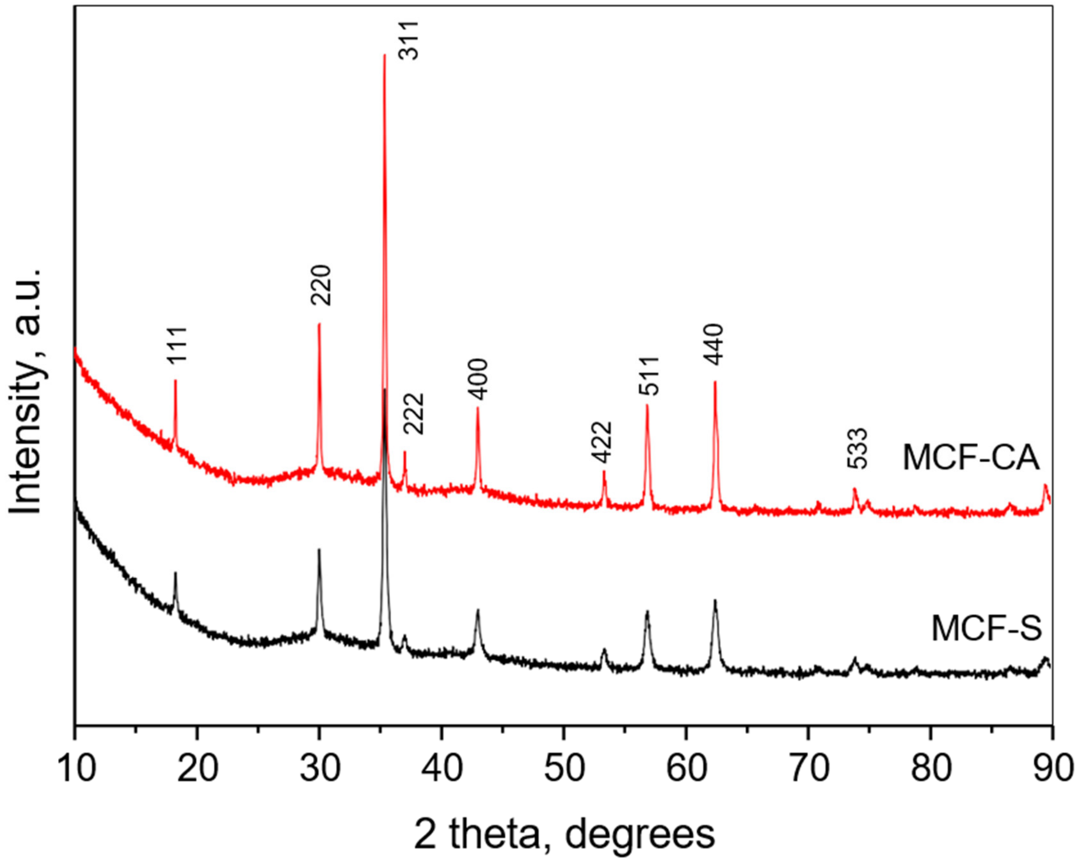

Figure S1. XRD patterns of MCF-S (black) and MCF-CA (red) after the thermal analysis up to 800°C. Crystallites size for MCF-S - 36 nm, unit cell parameter a=8.4163(8) Å, Crystallites size for MCF-CA – 77 nm, unit cell parameter a=8.4113(4) Å

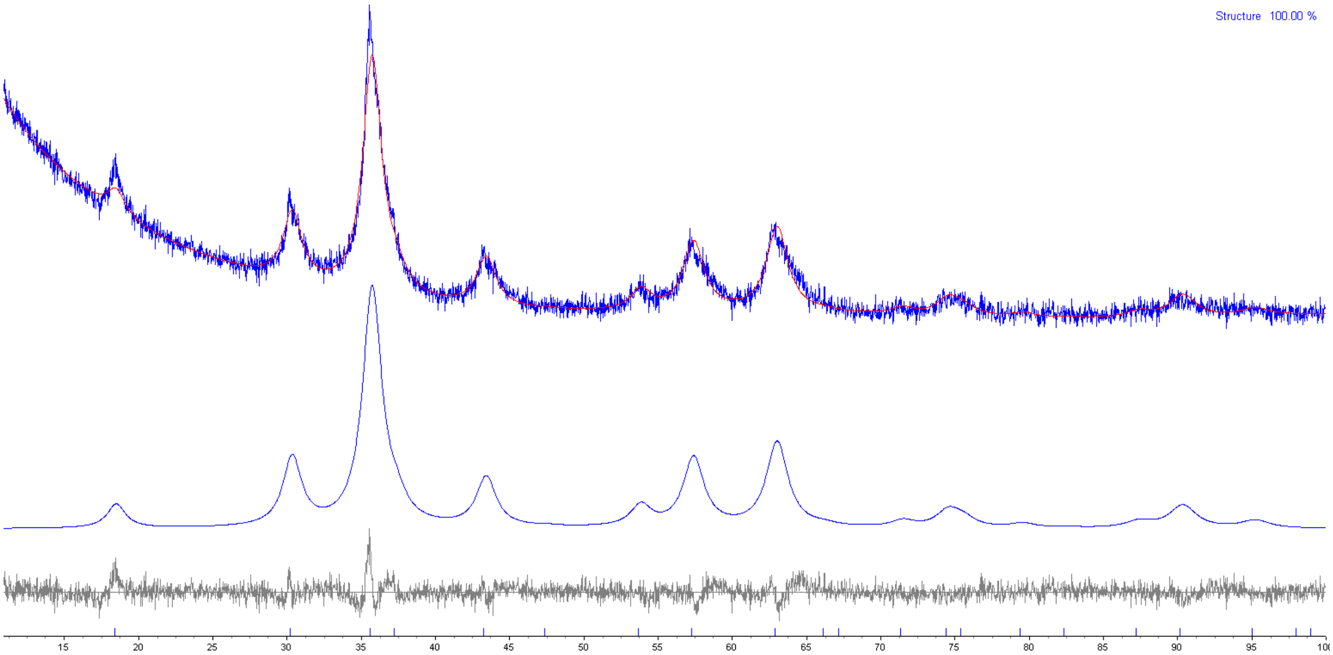

Figure S2. Rietveld plot of the MCF-S using one spinel phase. Upper blue line – experimental, red andlower blue line-calculated and grey line-difference plot.

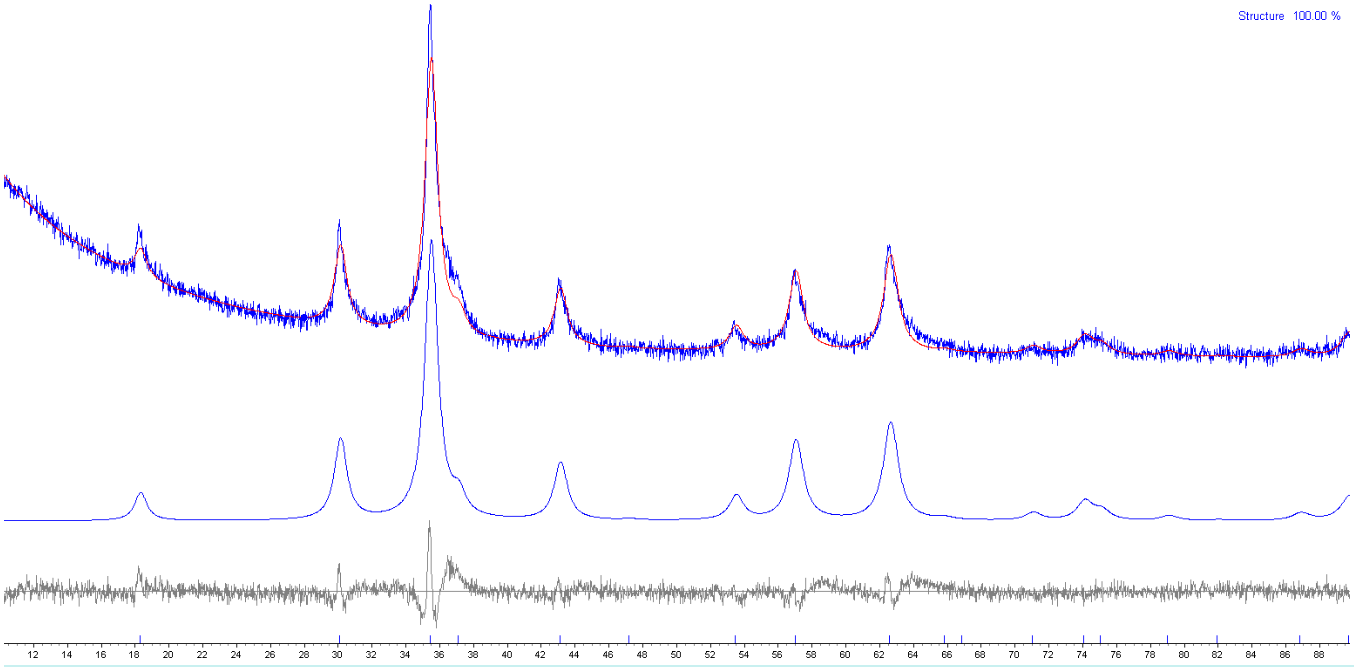

Figure S3. Rietveld plot of the MCF-CA using one spinel phase. Upper blue line – experimental, redand lower blue line-calculated and grey line-difference plot.

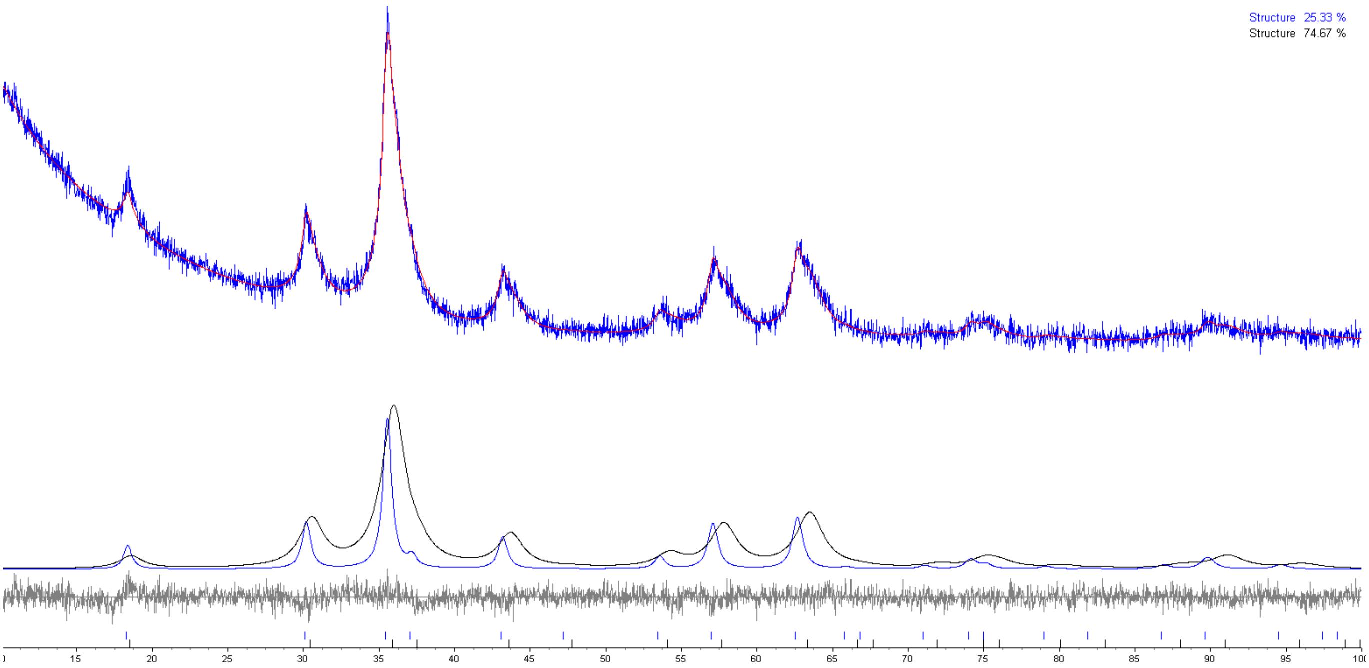

Figure S4. Rietveld plot of the MCF-S using two spinel phase. Upper blue line – experimental, red line – sum of the calculated, lower blue and black line-calculated and grey line-difference plot.

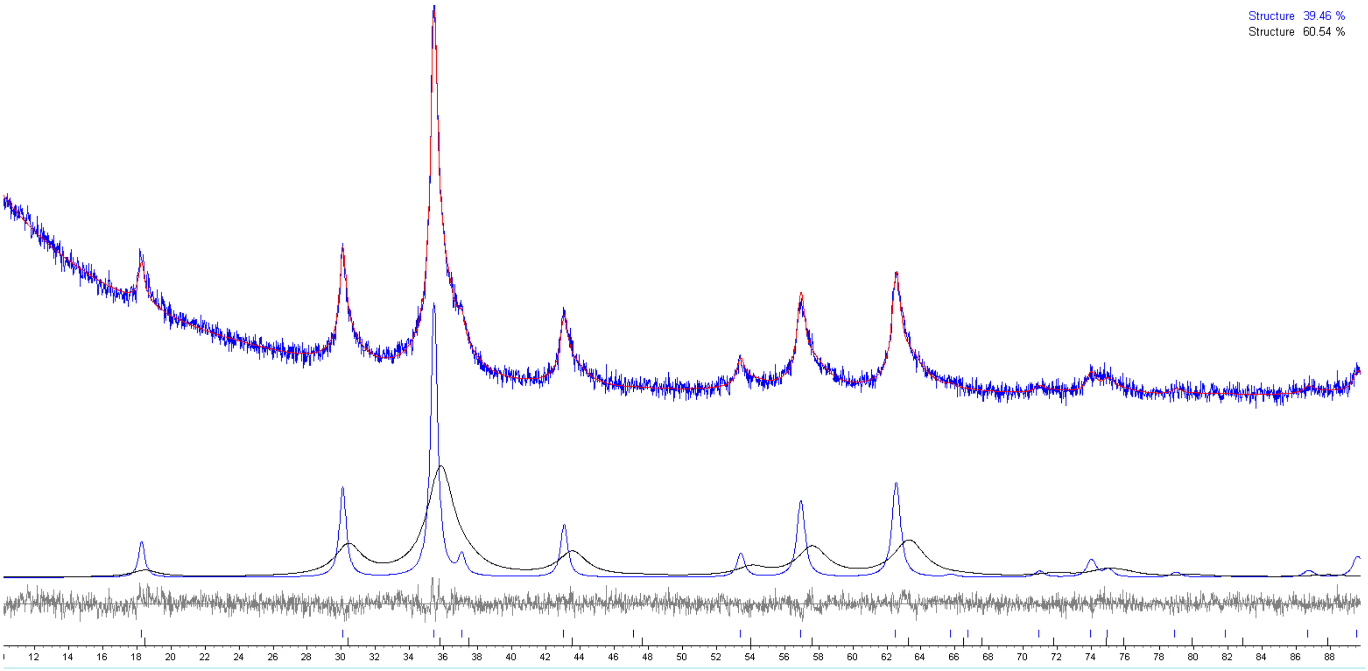

Figure S5. Rietveld plot of the MCF-CA using two spinel phase. Upper blue line – experimental, red line – sum of the calculated, lower blue and black line-calculated and grey line-difference plot.

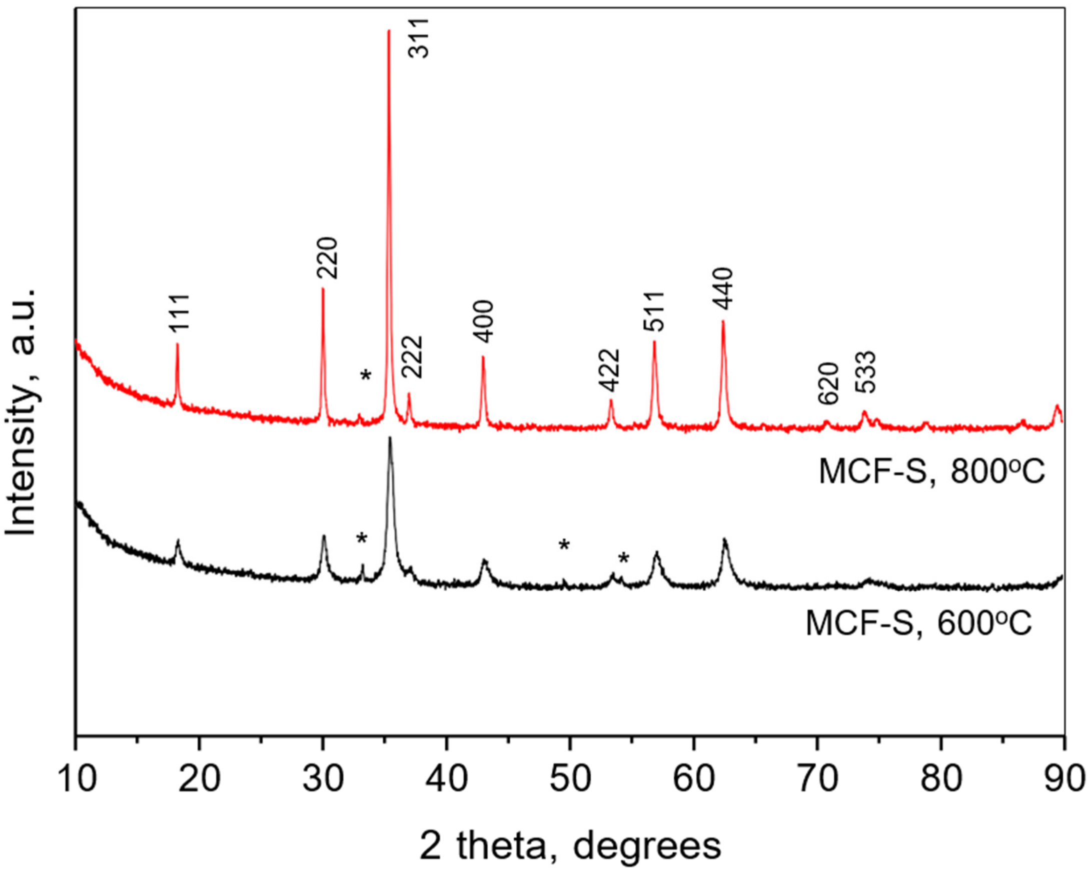

Figure S6. XRD patterns of MCF-S after the thermal treatment for 1h in air at 600°C (black) and at800°C (red). Asterix denotes hematite (Fe_2O_3) and/or bixbyite (Mn_2O_3). Crystallites size for MCF-S at 600°C-17 nm, unit cell parameter a=8.396(1)Å Crystallites size for MCF-S at 800°C – 55 nm, unit cell parameter a=8.4157(3) Å
